# Interventions to promote access to eyecare for non-dominant ethnic groups in high-income countries: a scoping review

**DOI:** 10.1136/bmjgh-2021-006188

**Published:** 2021-09-07

**Authors:** Lisa M Hamm, Aryati Yashadhana, Helen Burn, Joanna Black, Corina Grey, Matire Harwood, Roshini Peiris-John, Matthew J Burton, Jennifer R Evans, Jacqueline Ramke

**Affiliations:** 1 School of Optometry & Vision Science, Faculty of Medical and Health Sciences, The University of Auckland, Auckland, New Zealand; 2 Centre for Health Equity Training Research & Evaluation, University of New South Wales, Sydney, New South Wales, Australia; 3 Centre for Primary Health Care and Equity, University of New South Wales, Sydney, New South Wales, Australia; 4 Ingham Institute for Applied Medical Research, Liverpool, New South Wales, Australia; 5 School of Social Sciences, University of New South Wales, Sydney, New South Wales, Australia; 6 International Centre for Eye Health, London School of Hygiene & Tropical Medicine, London, UK; 7 School of Population Health, Faculty of Medical and Health Sciences, The University of Auckland, Auckland, New Zealand; 8 Performance Improvement, Auckland District Health Board, Auckland, New Zealand; 9 Moorfields Eye Hospital, London, UK

**Keywords:** review, eye diseases, health services research, public Health

## Abstract

**Purpose:**

People who are distinct from the dominant ethnic group within a country can experience a variety of barriers to accessing eyecare services. We conducted a scoping review to map published interventions aimed at improving access to eyecare for non-Indigenous, non-dominant ethnic groups residing in high-income countries.

**Methods:**

We searched MEDLINE, Embase and Global Health for studies that described an intervention to promote access to eyecare for the target population. Two authors independently screened titles and abstracts followed by review of the full text of potentially relevant sources. For included studies, data extraction was carried out independently by two authors. Findings were summarised using a combination of descriptive statistics and thematic analysis.

**Results:**

We screened 5220 titles/abstracts, of which 82 reports describing 67 studies met the inclusion criteria. Most studies were conducted in the USA (90%), attempted to improve access for Black (48%) or Latinx (28%) communities at-risk for diabetic retinopathy (42%) and glaucoma (18%). Only 30% included the target population in the design of the intervention; those that did tended to be larger, collaborative initiatives, which addressed both patient and provider components of access. Forty-eight studies (72%) evaluated whether an intervention changed an outcome measure. Among these, attendance at a follow-up eye examination after screening was the most common (n=20/48, 42%), and directly supporting patients to overcome barriers to attendance was reported as the most effective approach. Building relationships between patients and providers, running coordinated, longitudinal initiatives and supporting reduction of root causes for inequity (education and economic) were key themes highlighted for success.

**Conclusion:**

Although research evaluating interventions for non-dominant, non-Indigenous ethnic groups exist, key gaps remain. In particular, the paucity of relevant studies outside the USA needs to be addressed, and target communities need to be involved in the design and implementation of interventions more frequently.

**Key questions**

**What is already known?**

- Non-dominant ethnic groups living in high-income countries face worse eye health and vision outcomes compared to dominant ethnic groups.
- In high-income countries, most differences in eye health and vision outcomes between population groups are due to inequity.

**What are the new findings?**

- To date, most research about interventions to overcome inequitable access to eyecare for non-dominant ethnic groups has been conducted in the USA, targeting Black and Latinx Americans.
- Only about one-third of the studies reported being designed in collaboration with the target population; papers which were interconnected with wider research groups were more likely to work with communities than smaller research initiatives.
- Approaches which promoted at-risk patients to attend follow-up appointments after community screening have been subject to the most evaluation.

**What do the new findings imply?**

- The geographical scope of the research relevant to this topic is very limited; high-income countries outside the USA should investigate and address inequity across ethnic groups in access to eyecare.
- New initiatives implementing strategies to improve access to eyecare for non-dominant ethnic groups should start with genuine relationships (including collaborative design), have a plan for longitudinal involvement, and support empowerment of the target community beyond eyecare.

## Introduction

### Rationale

The United Nations’ Sustainable Development Goals aim to ‘leave no one behind’,^1^ however, many societal structures exist which systematically marginalise some groups while benefiting others.^2^ Specifically, allocation of resource tends to align with the interests, values, and norms of the ‘dominant’ group, being those with the highest concentration of wealth and decision-making power.^2^ In the context of Western high-income countries, dominant groups tend to align with the sociocultural norms and values of whiteness and neoliberalism. The establishment and maintenance of the socioeconomic gap between dominant and non-dominant ethnic groups is a reflection of structural racism.^2–4^ Here, we consider ‘race’ as a social construct which uses visual phenotyping and ancestry to justify systems of oppression and privilege, and ‘structural racism’ as the ways in which these systems of oppression and privilege (including education, criminal justice and health) are fostered.^4^ Pervasive structural racism in high-income countries means that non-dominant ethnic groups are often left behind,^5^ as evidenced most recently by mortality due to COVID-19 across the USA.^3^

Indigenous peoples worldwide have endured profound losses through colonisation and structural racism, as have many other non-dominant ethnic groups including Black,^3 5^ Latinx, Asian and Pacific Peoples.^4^ Since we report on Indigenous groups in a parallel report,^6^ here we focus on non-Indigenous, non-dominant ethnic groups (henceforth referred to as ‘non-dominant’). Health inequities exist on several fronts,^7 8^ including decreased access to preventative medicine,^9^ higher burden of disease^3^ and poorer access to assessment and treatment^10^ as well as palliative care.^11^ Similar trends are apparent within eyecare.^12–15^ Black and Latinx people in the USA are less likely than white people to report having visited an eye care provider.^16^ Seasonal migrant workers (typically from non-dominant ethnicities) spend long hours exposed to irritants which can cause infection, irritation or injury to the eyes,^17^ while having limited access to eyecare for resulting issues. Children from non-dominant ethnic groups are more likely to have unmet eyecare needs.^18^

Barriers to accessing eyecare are extensive, ranging from knowledge about eye conditions,^19^ capacity to cover primary and secondary costs, and a lack of trust in the institutions providing care. Although complex, barriers can be conceptualised through theoretical frameworks of access, such as that proposed by Levesque *et al.*^20^ This framework suggests that for a patient to move from identifying a healthcare need to having that need fulfilled, five steps are necessary, each of which requires medical providers to be accessible, and a patient to have the capacity to participate. From the provider perspective, Levesque *et al*^20^ argue that this includes: (1) approachability, (2) acceptability, (3) availability, (4) affordability and (5) appropriateness. On the demand side, patients need to be supported in their abilities to: (1) perceive, (2) seek, (3) reach, (4) pay and (5) engage. These categories are interdependent; for example, a noted barrier for non-dominant ethnic groups is a fear that seeking healthcare might compromise immigration status,^21^ this may reflect a decreased capacity to seek, and potentially poor provider approachability and acceptability. Similarly, communication barriers, where the provider does not share a patient’s language or cultural heritage^22 23^ could be conceptualised as an issue with acceptability, appropriateness or ability to engage. Notwithstanding the complexities, the framework is useful to identify barriers and consider ways to mitigate them.

### Aims

In this report, we sought to summarise published literature on interventions to promote access to eyecare for non-Indigenous, non-dominant ethnic groups living in high-income countries. The overarching aim was broken down into three questions:

1. What is the extent of the published literature?
2. What can we learn from reported effectiveness of interventions?
3. What can we learn from authors’ reflections on the potential to improve on interventions?

Similar questions have been raised by other researchers, but previous reviews have focused on improving access to eyecare in either low-income to middle-income countries^24 25^ or high-income countries,^26 27^ rather than exploring ethnicity-based inequities within a country. The goal of this, and our parallel paper,^6^ is to fill this gap for high-income countries.

## Methods

### Protocol and registration

The study protocol was published^28^; the protocol and this study are reported according to the Preferred Reporting Items for Systematic Reviews and Meta-Analyses extension for Scoping Reviews guideline.^29^

### Patient and public involvement

It was not feasible to include patient or public engagement in this review.

### Definitions and eligibility criteria

- Population: As outlined in our protocol,^28^ the target population is difficult to define. Self-identification of ethnicity is often fluid and nuanced,^30^ and appropriate terminology is actively debated.^31^ We opted for the term ‘non-dominant’, aligned with Benson’s definition that ‘non-dominant groups are minorities or even majorities that are unfairly marginalised in certain social contexts’.^32^ Since we addressed eyecare for Indigenous populations in a separate review,^6 33^ our target population for this review was limited to people who are not indigenous to the country where the study was conducted. We included studies in which at least 50% of participants were from a non-dominant, non-indigenous ethnic group.
- Intervention: We sought studies that described interventions to improve access to eyecare, according to the Levesque framework.^20^ We only included general health studies (eg, about diabetes) if there was sufficient data on eyecare to be a stand-alone resource.
- Setting: Studies needed to be conducted in high-income countries (as defined by the World Bank in 2019).^34^
- Study design: We included all studies which reported the impact of an intervention on participants, regardless of design, without a requirement for an evaluation of the intervention. Reviews, commentaries, editorials, and conference abstracts were excluded.
- Comparator: Studies with or without a comparator group were included. Studies with a comparator were considered ‘evaluated’.
- Other: We included all languages and publication dates.

### Information sources

An information specialist searched MEDLINE, Embase and Global Health databases as described^28^ on 28 July 2019 and updated on 26 August 2020. A sample database search is included online supplemental file 1, table S1.1.

10.1136/bmjgh-2021-006188.supp1 Supplementary data

### Selection of sources of evidence

All results from the search were entered into Covidence (www.covidence.org) for screening. Two authors (from LMH, JR, JB, CG and HB) independently reviewed each title and abstract to exclude those that did not meet inclusion criteria. Disagreements were resolved between the two reviewers, with a third author consulted when needed. The full text of each selected article was reviewed independently by two authors (from LMH, AY, HB, JR and JB), to determine whether they should be included in the data extraction phase. Again, conflict resolution was handled by discussion, and a third reviewer was asked to consult when needed.

### Data charting process

A data extraction form was developed based on the categories defined in our protocol^24^ and piloted by LMH, HB and JR. Simplifications which facilitated consistent data extraction are described below. Some proposed data extraction fields were eliminated after pilot extraction due to paucity, or inconsistent reporting of information (eg, socioeconomic status of participants). Data extraction was carried out independently for each publication by two authors (from LMH, RP-J, AY, JR, HB and JB).

#### Data items

##### What is the extent of the published literature?

###### Characteristics of the publication and targeted population

- Title, year, country.
- Targeted condition.
- Targeted ethnicity.
- Age: categorised as adult, child or all ages.

###### Characteristics of the intervention

- A free-text description of the intervention.
- Dimensions of Levesque framework addressed; this was recorded in ten binary entries, allowing the five dimensions of access to be assessed according to the patient and provider side independently (each included study contained between one and ten dimensions).
- Engagement with target population during development and implementation of the intervention (in most cases the study required a clear statement about community involvement, however, some publications were part of larger initiatives, so engagement with the target community was traced back through previous publications).

##### What can we learn from reported effectiveness of interventions?

- Study design: categorised according to types of comparator: none, sequential (pre–post) or concurrent (including alternate intervention or control).
- How many people participated.
- Free-text description of outcome measure.
- Whether the authors determined the intervention to be effective (this did not include an analysis of methodological quality).

##### What can we learn from authors’ reflections on the potential to improve on interventions?

- Free text describing authors’ reflections on strengths, weaknesses and recommendations.

#### Data synthesis strategy

##### What is the extent of the published literature?

We conducted a descriptive analysis of characteristics of studies and targeted groups, as well as of the characteristics of intervention strategies. Regarding targeted groups, if >50% of the participants were reported from one ethnic group, we reported that group as the target population. Since the terms used to refer to ethnicity varied across reports (eg, Hispanic, Latino) we assigned consistent names to each group which emerged during data extraction. Group names (such as ‘Black’ and ‘Latinx’) were based on our current understanding of best practice for promoting inclusivity and respect, however, we recognise the drawbacks of their use; these terms reflect norms from specific contexts at this time, and do not capture the diversity of cultures and people within each category. Although most studies specifically targeted one ethnic group, some studies targeted a community-based factor other than ethnicity (eg, recent immigrants from varied backgrounds). If our inclusion criteria were still met (>50% non-dominant, non-Indigenous participants), but no single ethnic group targeted (eg, if the breakdown of participants was 25% Pacific Peoples, 25% Latinx and 25% Black) we used the term ‘mixed ethnicities’, to reflect the mixture of ethnic backgrounds targeted. Regarding interventions, we summed binary data from the Levesque framework section to categorise interventions as patient focused, provider focused or both. We also plotted author collaborations to understand how studies were linked within collaborative networks.

##### What can we learn from reported effectiveness of interventions?

We limited our analysis in this section to studies with a comparator, and grouped them by outcome measure. If a study reported multiple outcomes across several papers, we reported each. However, if several outcomes were reported in a single paper, we used the outcome closest to behaviour (eg, attendance at a follow-up eye examination was recorded rather than knowledge about target condition, for cases in which both these outcomes were reported). We simplified descriptive text about outcome measures into the following categories:

- Survey (results from an assessment of health knowledge or perspectives about accessing eyecare).
- Attendance at screening.
- Attendance at follow-up eye exam (percentage or referred participants who attended a follow-up eye exam after screening—including self-reported and confirmed attendance, and all time frames).
- Adherence to (non-attendence) recommendations (including treatment and prevention measures).
- Health (eg, incidence of blindness, vision impairment, control of diabetes, etc).

We did not assess the quality of evidence, rather, we relied on the authors notes about the comparator, outcome measure and results, and categorised effectiveness as follows:

- Effective (improvement in outcome measured with statistical significance).
- Inconclusive (improvement in outcome measured but not reaching statistical significance, or a negative result but authors note a design flaw may have masked an important finding).
- Ineffective (no change in outcome measure, or a change in the opposite direction).

##### What can we learn from authors’ reflections on the potential to improve on interventions?

To address the third question, a thematic analysis was conducted across all free-text (qualitative) data fields including authors’ reflections on strengths, weaknesses and recommendations within each identified paper, to converge on perceived elements associated with ‘success’. We used an inductive approach to coding,^35^ where iterative use of four phases of analysis (data familiarisation, generating initial codes, categorisation and emergence of themes, and reviewing of identified themes) was applied to qualatative data. A single author completed the analysis manually by categorising free-text data fields using an Excel spreadsheet and colour coding techniques to allow for thematic synthesis of data. We were interested in understanding the ‘weight’ of each theme across the included studies, and therefore, also captured frequency (eg, the number of papers that referred to an identified theme). The themes that were most referenced across the set of papers as a whole, are presented in the results section.

### Grey literature

Although not planned,^28^ in response to review we checked whether additional initiatives were highlighted in unpublished literature (details of our grey literature search are available in online supplemental file 1, table S1.2). Unpublished initiatives surfacing from this additional search are summarised at the end of our results section, to provide additional context for our aims.

## Results

### Summary of sources of evidence

**Figure 1:**
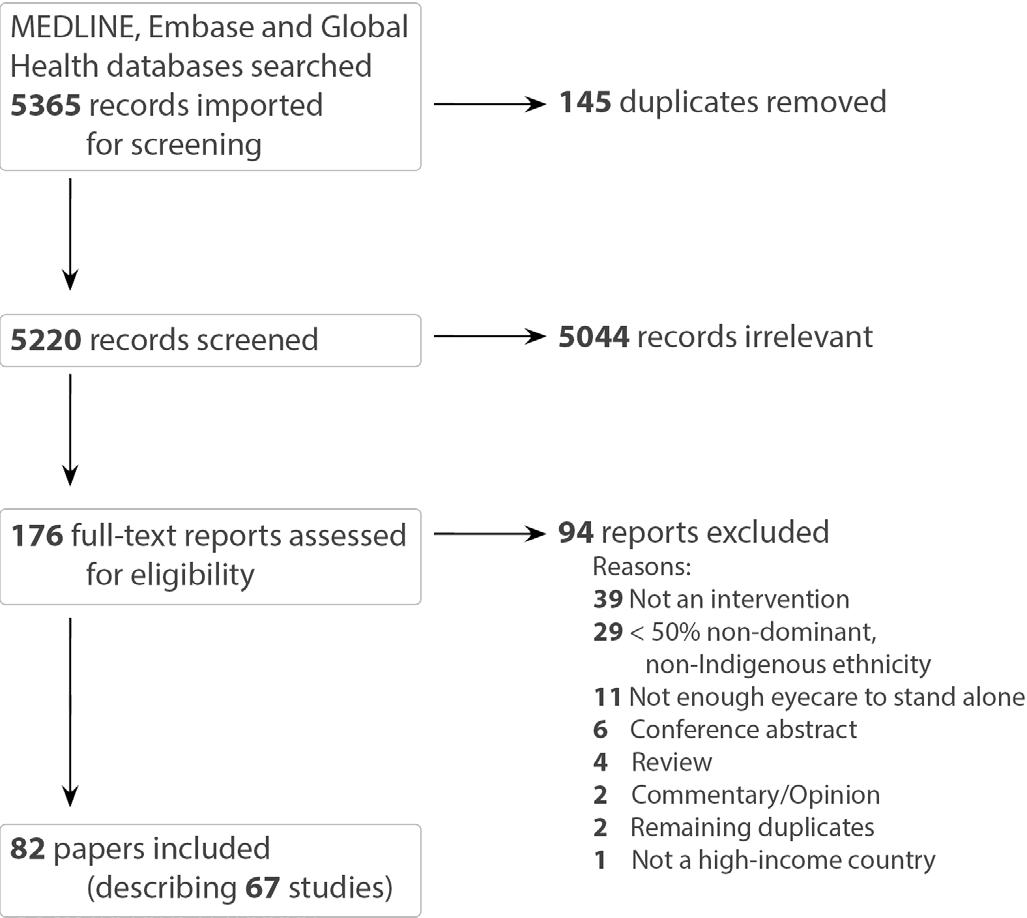
PRISMA flow diagram. PRISMA, Preferred Reporting Items for Systematic Reviews and Meta-Analyses.

Our search of the published literature identified 5220 unique records which were screened by title and abstract, and 176 were included in the full text screening. Eligible references included 82 papers, describing 67 studies (figure 1).

### What is the extent of the published literature?

#### Characteristics of publication and targeted groups

**Figure 2:**
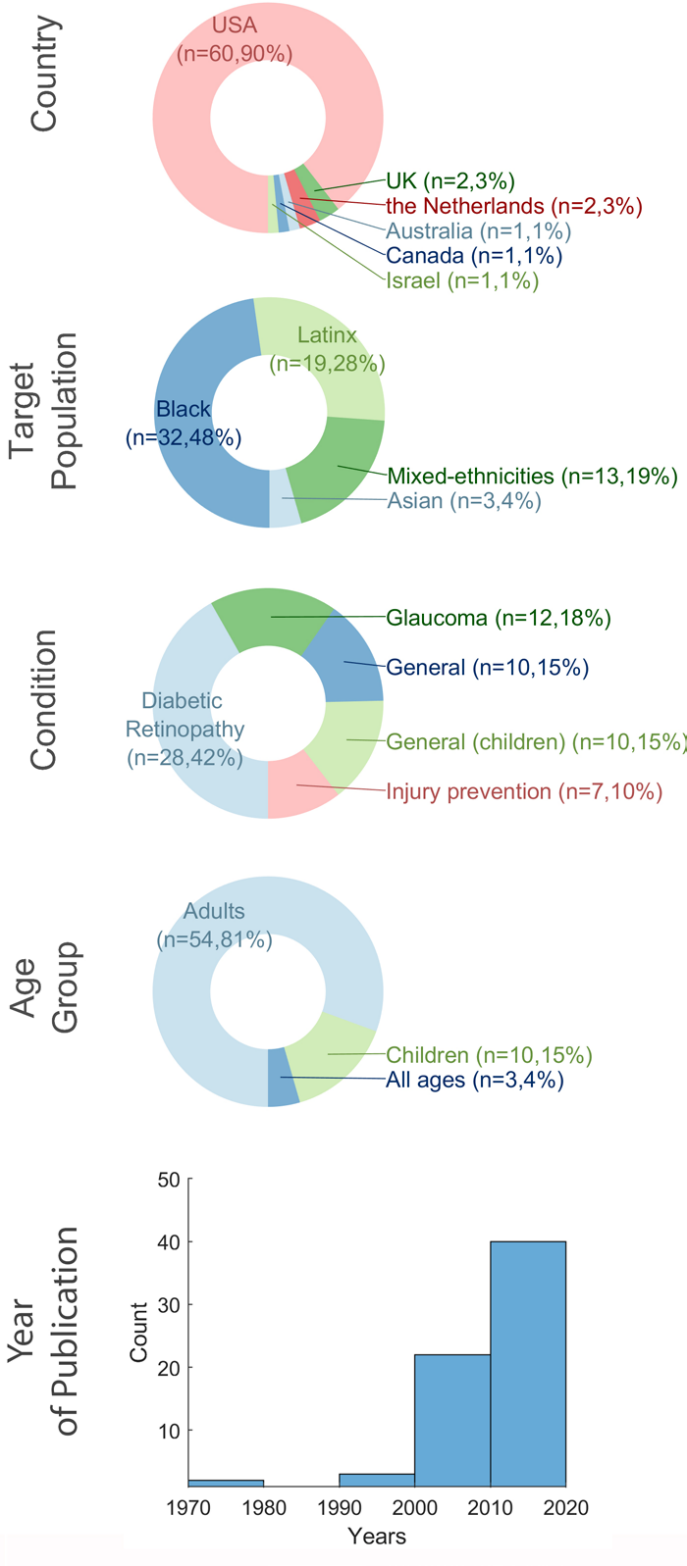
Characteristics of publications (studies) and target groups.

Characteristics of publications and targeted groups are summarised in figure 2. The majority of research (n=60, 90% of studies, or n=75, 92% of papers) was conducted in the USA, targeting Black or Latinx Americans. Two studies from the UK^19 36^ and one from Canada^37^ sought to improve eyecare for Asian populations. Research in The Netherlands,^38 39^ Israel^40^ and Australia^41^ did not focus on a specific ethnic group, rather each study aimed to improve access for non-dominant ethnicities more generally. Studies focused on adults targeted diabetic retinopathy (n=28, 42%), glaucoma (n=12, 18%) and injury prevention for seasonal farm workers (n=7, 10%). Twenty studies promoted access for general eye health, some for adults or all ages (n=10, 15%), and some specifically for children (n=10, 15%). Most papers were published in the last 20 years (n=60, 90%), with a spike in last 10 years (n=40, 60%), driven by work done in the USA.

#### Characteristics of the interventions

**Figure 3:**
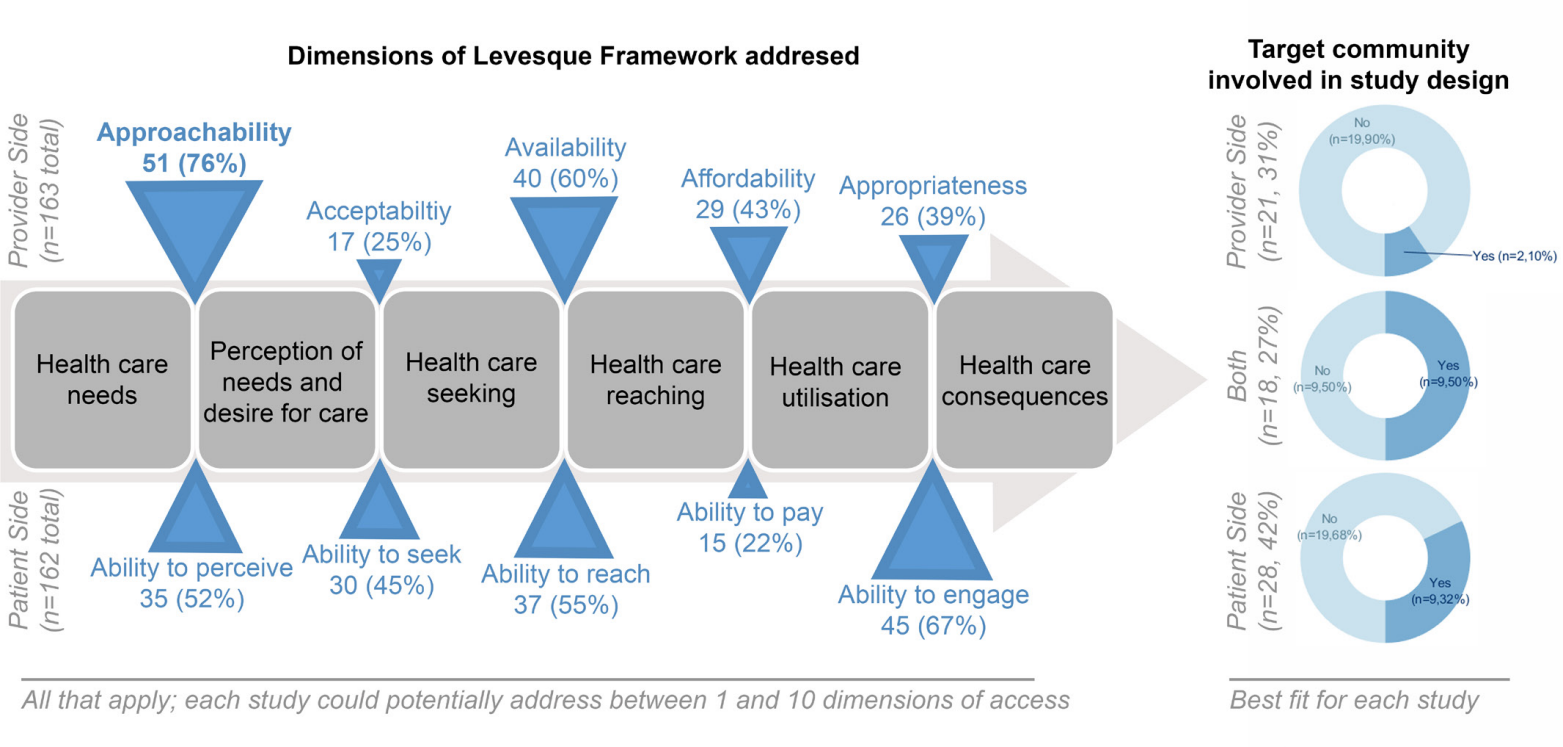
Characteristics of interventions, in terms of dimensions of access addressed and involvement of target community in study design. For the Levesque framework, a single study typically addressed more than one dimension of access (the denominator for each is the total number of studies). When studies were categorised as mainly addressing patient, provider or both sides of access, those which targeted both were most likely to have engaged with the target community.

Figure 3 summarises the characteristics of the interventions. Most studies (n=59, 88%) addressed more than one dimension of access, with most emphasis on making services more approachable (n=51, 76%, eg, running screening programs in convenient locations), and least emphasis on making eyecare more acceptable (n=17, 25%, eg, training culturally sensitive clinical staff)^42^ and improving the patient’s ability to pay (n=15, 22% eg, providing financial incentives).^43^

Studies were relatively evenly split between focusing on the patient side of access (n=28, 42%), provider side (n=21, 31%) and integrating both (n=18, 27%). Approaches which focused on the provider side of access mostly described community screening (n=15, 71%) and comprehensive community eyecare services (n=3, 14%), whereas approaches focused on the patient side of access described health education (n=16, 55%), and supporting people to access services (n=13, 45%, including reminder calls and one-on-one counselling to identify and overcome barriers to access). Only 20 studies (30%) reported having the target community involved in the design and implementation. Studies that involved the target community in the design were more likely to address both the patient and provider sides of access. Very few studies which engaged with the community ran isolated screening or service programmes.

**Figure 4:**
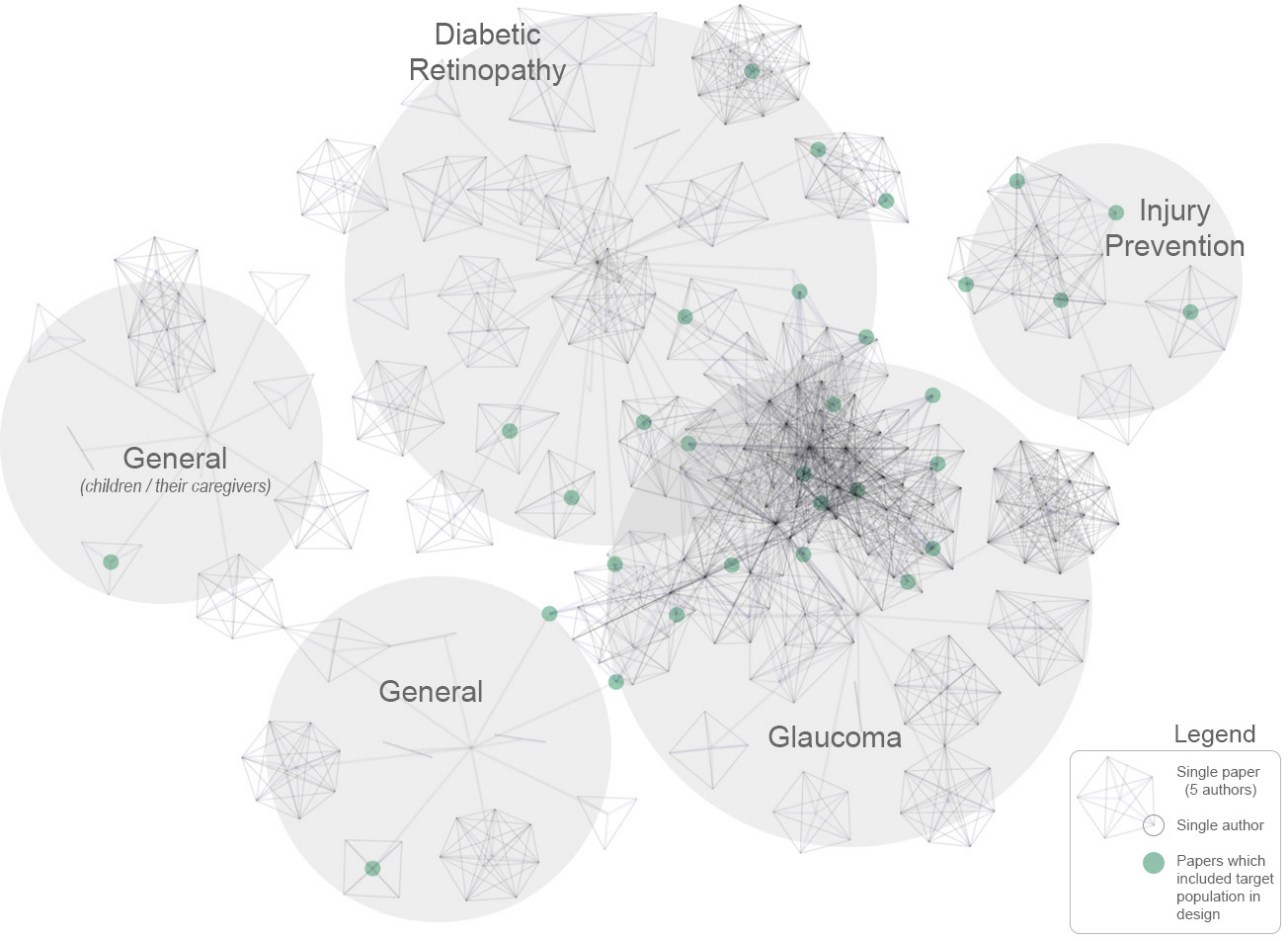
Summary of included publications by relationship between authors and conditions. Each node represents an author, and each cluster of nodes represents a paper, or groups of papers by similar authors. Papers which included the target population in the design are highlighted in green. Note the clustering of green dots within collaborative networks (although with notable exceptions).

For each paper, we recorded the number of other included papers connected through coauthorship, as well as the strength of the connection between papers (sum of common authors). Papers which involved the target community in the study design had more connections to other papers, and stronger connections between papers than those that did not (both p<0.001). In other words, studies that involved the target community in the design were also more likely to originate within wide collaborative networks than more isolated initiatives. Figure 4 allows visualisation of collaborative networks by plotting authors by their connections to papers and targeted conditions (those which engaged with the target community in the design are highlighted in green).

### What can we learn from reported effectiveness of interventions?

Most studies (n=48, 72%) included a comparator; of these, 20 (42%) measured an outcome before and after an intervention, while 28 (58%) compared alternative interventions concurrently. A summary of interventions, grouped by the comparator type, can be found in the online supplemental table S1.2. In this section, we summarise studies with a comparator, organised by outcome measure (surveys: 9/48, 19%, screening: 5/48, 10%, eye exam: 20/48, 42%, non-attendance adherence: 8/48, 17%, health: 6/48, 13%).

10.1136/bmjgh-2021-006188.supp2 Supplementary data

#### Survey of knowledge

Implementation of a variety of educational programmes, including glaucoma^44^ diabetic retinopathy^45–47^ and general eye health for adults^44 48^ and children^49^ all resulted in improvements in knowledge (although not all statistically so), as did writing out instructions for glaucoma medications.^50^ Although these interventions improved knowledge, most authors highlighted the gap between self-reported knowledge, and a change in behaviour or action.^19 46 48 51^

#### Improving attendance at screening

Five studies specifically quantified whether attendance improved as a result of an intervention, all reported improvements in screening rates after initiatives were launched.^41 52–55^ Recruitment initiatives^41^ and automated reminder calls^56^ were both highlighted as effective strategies to improve attendance.

#### Improving attendance at follow-up eye exams

Compared with measuring knowledge and attendance at screening appointments, studies which measured attendance at a follow-up eye exam were more likely to be inconclusive (n=6/19, 32%) or ineffective (n=2/19, 11%). Specifically, education programmes, although helpful to improve knowledge on surveys, showed limited success for improving attendance at follow-up eye exams,^57–59^ and having patients sign a contract stating that they will attend an appointment was ineffective.^60^

Studies implementing supportive reminder calls were more frequently considered effective^61–63^ than improving education or having patients sign contracts, however, not universally so. For example, Meng *et al*^64^ used reminder calls in a wider context (in an attempt to reduce ethnic disparities in Medicaid use) and found it was not effective for most groups. The nature of the supportive interaction appeared more important than whether or not the interaction took place.^63 65–67^ For example, ‘behavioural activation’ (which includes a trained support person helping a patient to identify, and working through, barriers) was an effective approach in several included studies.^65 68–71^ A study about Black Americans at risk of diabetic retinopathy^65^ (described across three papers^65–67^ showed behavioural activation to be more effective than more general supportive therapy (a placebo treatment where participants are provided one-on-one empathetic discussion about the impact of diabetes on their lives, in a comfortable environment). ‘Patient navigators’ likely fill a similar role to those who facilitate behavioural activation; they help guide patients through the steps of eyecare, providing support as needed. An initial randomised controlled trial (RCT) evaluating effectiveness of adding patient navigators within an already supportive eyecare programme showed inconclusive results^72^ but a follow-up RCT is underway.^73–75^ Hiring school nurses to help students navigate from screening to full eye exams and comply with treatment was reported to be effective compared with screening alone^76^ (although some universal childhood screening programmes were very effective at promoting follow-up).^77^

Initiatives with wider, integrated approaches also demonstrated effectiveness at improving attendance at follow-up eye exams (though not always significantly so). These initiatives tended to include several components, such as prescheduling follow-up appointments,^43 78^ running follow-up appointments at convenient locations,^79^ incorporating education elements^43 59 80^ and providing financial incentives.^43 80^

#### Improving adherence with preventative measures or treatment

Several studies showed that providing appropriate, free eyewear helps to promote use of protective eyewear among seasonal farm workers,^81^ though training respected peer-workers to provide education about eye health,^17 82^ and to model use of protective eyewear 81–85 was more effective. Phone messages to remind workers to use protective eyewear also appeared to be an effective strategy to increase use.^86^

For mixed-ethnicity glaucoma patients, prerecorded translations were deemed equally effective as having translators available to enable visual field tests,^40^ and educational cartoons (designed to span language and cultural barriers) effectively improved adherence to amblyopia treatment.^38 39^

#### Improving health

Community low vision services improved self-reported functional vision (using a premeasure/postmeasure),^87^ and an educational programme for seasonal farm workers led to a reduction in self-reported eye pain.^17^ Measured, rather than self-reported, health outcomes were more difficult to improve with interventions, however, three preliminary studies aimed at reducing glycated haemoglobin (HbA1c) levels among people with diabetes showed encouraging results (one focused on building trust between patients and ophthalmologists,^42^ another on supportive interactions with community health workers^70^ and another with an integrative web-based diabetes management system.^88^

Only one study included in this review reported incidence of blindness as an outcome. Baker *et al*^89^ described a comprehensive, integrated programme (addressing several dimensions of access) which supported people with diabetes, living in low-income areas within Houston, USA (53% Black, 30% Latinx). In 1986, just prior to initiation of the programme, the incidence of blindness in this community was 9.5/1000, but it reduced to 2.7/1000 by 1989 as access to eyecare improved. Although the authors note that the confidence intervals do not indicate the change was statistically significant, clinically, they report the reduction in blindness to be very encouraging.

### What can we learn from authors’ reflections on the potential to improve on interventions?

Thematic analysis of the strengths, weaknesses and recommendations highlighted within each study resulted in several themes, the three most frequently noted are described briefly below.

#### Relationship

The most commonly noted factor attributed to the success of an intervention was a genuine relationship between participants and staff of the initiative.^17 42 49 61 65 69 72 82–84 90–93^ For studies not collaborating with communities from the start, any social or culturally sensitive, aspects of the intervention tended to be highlighted as an important aspect of the project.^38 39 44 52 70 71 94^ Although cultural concordance was an important factor, one study noted that rapport can be equally effective.^69^ A related concept was that strategies effective in one community, or non-dominant ethic group, were not always effective in another^41 56 64^; communities are unique, and each local community should be involved in the development of interventions targeting them.

#### Coordination

Another commonly noted idea was that a coordinated, longitudinal approach was key to successful initiatives.^43 51 52 54 61 72 76 89 95–104^ Often this meant a branch of the project was dedicated to managing participants’ transition from screening through to treatment and ongoing follow-up. It was noted by several studies that this type of approach required more resources than short, isolated interventions; both in terms of funding^52 54 61^ and buy-in from many different professional staff.^43 102 104^ A coordinated approach also meant being ready to treat a wide range of conditions. For example, prompt provision of refractive error and scheduling of cataract removal, as part of a diabetes intervention, was highlighted as more effective than having a limited, condition-specific scope.^103^

#### Social gradient of target community

Several authors noted that interventions to improve access to eyecare were limited by socioeconomic and cultural needs of target communities,^36 43 57–59 64 79 91 105^ suggesting the impact of underlying structural racism and generational poverty. The reliability of contact details appeared to be a common example of deeper socioeconomic issues^36 64 91 105^; those most at-risk of poor eye health were noted to have limited phone data, less stable housing and at times exhibit a reluctance to provide accurate identifying information (for fears related to immigration status, or being billed for help received).^79^ Poor literacy was thought to limit the impact of some interventions directly,^58 105^ and broader inequity across the social gradient (including education, employment and income) reflected the dimensions of access least addressed; the shortage of culturally concordant ophthalmologists was noted as a barrier to enhancing acceptability of services,^43^ and socioeconomic disadvantage limited patient’s ability to pay for perceived or real costs, limiting access.^57^

### Grey literature

The grey literature search identified 624 potential resources. Many of these highlighted the disproportionate burden of vision impairment among non-dominant ethnic minorities and the need for, planned, or newly established eyecare infrastructure to promote equity. Ultimately, we identified twelve resources that described initiatives to promote access directly, highlighted below (links in online supplemental file 1, table S1.3).

In the USA, the Affordable Care Act, the National Eye Institute, the American Academy of Ophthalmology, the National Association of Hispanic Nurses and some drug companies offer targeted education, and sometimes screening and treatment services (eg, EyeCare America, Travatan Project Focus, Partnership for Sight Initiative). Some local health boards (eg, in San Joaquin) and other initiatives (eg, Ventanillas de Salud and Juntos por la Salus) specifically target Latinx people living in the USA with health promotion programmes to promote access to healthcare, including eyecare. In Europe, initiatives in Glasgow and Wales appear more integrated, and a project in Finland focused on strengthening the provider side of access. The initiative in Finland was the only one we found which reported specific outcomes, notably that number of delayed follow-up eye appointments decreased dramatically after implementation.

Our grey literature search also highlighted more academic research from the USA underway, for example, the Screening and Intervention for Glaucoma and Eye Health through Telemedicine Programme.

## Discussion

### Summary of evidence

#### What is the extent of the published literature?

Most studies were conducted in the USA (90%), attempting to improve access for Black (48%) and Latinx (29%) populations. Issues with inequitable access to eyecare have been documented for high-income countries outside the USA (eg, in the UK,^106^ New Zealand^107^ and Sweden,^108^) yet, knowledge of the inequity and barriers does not appear to have translated to research about interventions to address them.

There were some similarities between this and our parallel review that synthesised eyecare service delivery models targeting Indigenous populations.^6^ Both reported a narrow geographic scope (USA: 90% in this review vs Australia: 67%^6^), both noted a focus on diabetic retinopathy (45% vs 53%^6^), and both noted that only about 30% of studies engaged communities in the design process. There were also some key differences; for example, glaucoma was targeted more for non-Indigenous, non-dominant groups (18%) than for Indigenous peoples (2%).^6^ Interventions focused on trachoma appeared specific to Indigenous peoples in Australia^6^ (as this is not prevalent in other populations), whereas eye irritation/injury experienced by seasonal farm workers was only highlighted for non-dominant, non-Indigenous populations in the USA. The existence of trachoma in Indigenous Australia and worker risk in the USA reflects the failure of high-income countries to meet basic environmental and social needs (including sanitation and workplace safety) of routinely marginalised people. That we identified some response to these disparities in the literature is encouraging, but more and better evidence is required to improve the eye health of these populations.

#### What can we learn from reported effectiveness of interventions?

Of the studies which reported at least one comparator, 40% used attendance at a follow-up eye exam after screening as the primary outcome measure. Interventions which improved attendance at follow-up tended to include personalised, supportive interactions between patients and providers, designed to help patients identify and address barriers to attendance. The approaches to improve access to eyecare highlighted here are similar to those summarised in other populations. A recent systematic review found tele-screening is likely cost-effective, especially in low-income areas.^26^ A systematic review of interventions to improve attendance for screening and follow-up for diabetic retinopathy also concluded that ‘behavioural change techniques’ (aimed at the patient), and quality improvements (aimed at the provider) were effective at improving attendance. They found that on average attendance increased by 12% compared with no intervention, but noted that communities with lower baseline levels of attendance benefited the most from intervention.^27^ Indeed, several studies highlighted in this review showed improvements over 20%.^52 61 66 68^

It is important to note that focusing on behavioural change can decrease health outcomes, if it is at the cost of considering the complex social gradient in which non-dominant communities exist.^109^ Several larger initiatives captured in this review appeared to appreciate the risks of putting the onus on already marginalised patients. These studies tended to weave elements of behavioural activation within more comprehensive services, including convenient community screening, streamlining referral and treatment services including prompt booking of required appointments in convenient locations, culturally concordant staff and financial incentives. Although there is limited information about health outcomes captured in this review, one encouraging study in the USA, targeting urban, low-income, mostly Black patients with diabetes demonstrated a decrease in the incidence of blindness as a result of an intervention targeting several aspects of access in a coordinated manner.^89^

#### What can we learn from authors’ reflections on the potential to improve on interventions?

Authors’ reflections emphasised the need for enabling relationships between provider and patient, coordination of steps from screening through to treatment and the need to address wider issues of poverty and inequity in health and education. The first two can be addressed through improved interventions, but the latter requires wider systemic change. Programmes to enhance ethnic diversity in ophthalmology programmes^110^ is an example of how institution-level changes could improve currently hard to address aspects of access. However, these programmes will take time to have an impact, as will the further steps of addressing poor representation in ophthalmology leadership,^111^ and structural inequity more generally.

#### Considering all three questions together

Despite authors noting that genuine relationships between patients and providers is a key element to success, only 30% of the studies involved the target community as part of the design process. Similarly, despite author reflections on the importance of consistent, longitudinal initiatives, most published projects were of limited scope and duration. Studies which achieved both these targets were generally housed within ongoing highly collaborative initiatives, delivering interventions which prioritised empowering patients while providing better services in a coordinated way. However, studies within these larger initiatives were less likely to report an effective study outcome than more isolated studies (despite larger studies being evaluated at a slightly higher rate). This appeared to be because the larger initiatives took more flexible, longer-term approaches, tended to target the most hard-to-reach communities, and focused on more meaningful outcomes (eg, attendance at follow-up eye exam rather than knowledge on a survey), which are harder to achieve. Taken together, appropriate measures of impact are an important area of future research, perhaps frameworks such as reach, efficacy, adoption, implementation and maintenance^96^ start to address this issue. There are additional unpublished initiatives which provide education, screening and treatment to promote equitable access to eyecare, initiated from within various sectors, but few appear to be resourcing at-risk communities to provide solutions, or highlighting community-driven initiatives.

### Study limitations

Several definitions or concepts were simplified to facilitate a usable summary, and some categorisations were subjective. In particular, the population of interest (as stated in the Methods section, and the protocol^28^) is difficult to define. Future work may benefit from broadening the target population to look at those with low socioeconomic status in high-income countries or, alternatively, narrowing the target population to look at a specific non-dominant ethnic group in a specific place. The results of our review suggest the bulk of existing research is focused on Black people living in low socioeconomic areas of USA, which may not generalise to other communities in other high-income countries (with different politics, experience of structural racism and eyecare systems).

Many studies noted reliance on church groups, not-for-profit support networks, or community boards to develop, guide and support the initiatives described in publications. Collaboration with these community programmes (often setup to support specific, but not necessarily ethnicity-defined groups, such as recent immigrants, homeless populations or those living in poverty) appeared integral for successful interventions. These types of initiatives should be further explored, as there are likely to be many which are unpublished. Even the grey literature did not capture grassroots initiatives, rather, relevant resources appeared to be press releases from larger associations.

## Conclusion

In this scoping review, we have mapped published interventions aimed at improving access to eyecare for non-dominant ethnic groups. Most interventions were carried out in the USA, targeting Black and Latinx communities, and described screening programmes targeting people with diabetic retinopathy and glaucoma. More research is needed to address the inequities in access to eyecare in high-income countries beyond the USA. Only 30% of studies included the target population in planning and implementation, the majority of which were larger, coordinated initiatives. Most evaluated interventions focused on improving attendance at a follow-up eye exam after screening; among these, personalised support to help patients address barriers to attendance appeared most effective. However, placing onus on patient behavioural change has limits, and several papers suggested that inequity in eyecare accessibility will persist if structural racism and generational poverty are not overcome. While striving to address underlying socioeconomic and cultural needs, this review suggests that building genuine relationships between patients and providers (including collaborative intervention design) and establishing longitudinal coordinated initiatives (which address many barriers to access simultaneously) are important starting points.

### Dissemination statement

The scoping review outlined here is part of a larger study to improve access to eyecare services for Indigenous and non-Indigenous ethnic groups in Aotearoa/New Zealand. The findings will be useful to policymakers, health service managers and clinicians responsible for eyecare services in New Zealand, as well as in other countries with similar marginalised population groups. We will develop an accessible summary of the results for posting on institutional websites and dissemination at stakeholder meetings.

## Data Availability

All data relevant to the study are included in the article or uploaded as online supplemental information.
