# Supplementary material for: Interventions to promote access to eyecare for non-dominant ethnic groups in high-income countries: a scoping review
Source: BMJ Glob Health. 2021 Sep 7;6(9):e006188. doi: 10.1136/bmjgh-2021-006188 (PMC8424858; doi:10.1136/bmjgh-2021-006188)
Supplement: Supplementary data [file bmjgh-2021-006188supp001.pdf]

Supplementary File 1

Table S1.1. *Published Literature Search strategy - example for one database*

1 exp Ophthalmology/ (21885)
2 Optometry/ (5403)
3 ((eye$ or ocular or vision) adj2 (care or health or service$)).tw. (6849)
4 visual acuity.tw. (59866)
5 (ophthalm$ or optomet$).tw. (100106)
6 exp Eye Diseases/ (543545)
7 (glaucoma$ or ocular hypertension or cataract$).tw. (103115)
8 ((diabet$ or proliferat$) adj3 retinopath$).tw. (23455)
9 (diabet$ adj3 (eye$ or vision or visual$ or sight$)).tw. (4324)
10 (retinopath$ adj3 (eye$ or vision or visual$ or sight$)).tw. (1996)
11 (dilated adj2 fundus).tw. (561)
12 (retinal adj2 exam$).tw. (1497)
13 (myop$ or hyperop$ or hypermetrop$ or anisometrop$ or ammetrop$ or astigmati$ or presbyop$).tw. (60420)
14 (refractive adj1 error$).tw. (8930)
15 Eyeglasses/ (7396)
16 (spectacle or spectacles or glasses).tw. (17268)
17 (eyeglasses or eye glasses).tw. (793)
18 or/1-17 (676343)
19 Ethnic Groups/ (58731)
20 Minority Groups/ (13034)
21 ((ethnic$ or racial$ or cultur$) adj3 (group$ or minorit$ or population$ or diverse$ or origin$)).tw. (79505)
22 ((ethnic$ or racial$ or cultur$) adj3 (inequalit$ or inequit$ or disparit$ or equit$ or disadvantage$ or depriv$)).tw. (12388)
23 ((population$ or communit$) adj3 (divers$ or disadvantage$ or depriv$)).tw. (31840)
24 non-Indigenous.tw. (1904)
25 (visible adj1 minorit$).tw. (179)
26 Refugees/ (9393)
27 "emigrants and immigrants"/ (10951)
28 (migrant$ or immigrant$ or emigrant$ or refugee$).tw. (46175)
29 asylum seeker$.tw. (1373)
30 Urban Population/ (56887)
31 urban poor.tw. (696)
32 Cultural Characteristics/ (15856)
33 Cross-Cultural Comparison/ (24923)
34 Cultural Diversity/ (10999)
35 Cultural competency/ (4994)
36 Cultural deprivation/ (1142)
37 exp African Continental Ancestry Group/ (82727)
38 exp Asian Continental Ancestry Group/ (67897)
39 Continental Population Groups/ (20766)
40 (Afr$ adj2 American$).tw. (51367)
41 (Afr$ adj2 Caribbean$).tw. (2292)
42 (west adj2 (india$ or indies)).tw. (4801)
43 (American$ adj1 black).tw. (2053)
44 (Asian$ or Indian$ or Pakistan$ or Bangladesh$ or Bengal$).tw. (181277)
45 Pacific.tw. (31669)
46 (Hispanic or Latino or Latin American$ or Puerto Ric$ or Mexican$).tw. (81920)
47 or/19-46 (667128)
48 Program Evaluation/ (59786)
49 program$.tw. (809937)

50 ((educat$ or behaviour$ or behavior$ or improv$) adj3 (intervention$ or activat$)).tw. (76474)
51 Delivery of Health Care/ (84907)
52 Health Services Accessibility/ (69393)
53 Patient Acceptance of Health Care/ (42735)
54 Health Promotion/ (69909)
55 (health adj2 (promotion$ or knowledge or belief$)).tw. (39822)
56 Health Education/ (59039)
57 (educat$ adj2 (information or material or leaflet)).tw. (6826)
58 Health Knowledge, Attitudes, Practice/ (103696)
59 Patient Education as Topic/ (82258)
60 Persuasive Communication/ (3553)
61 "Surveys and Questionnaires"/ (429592)
62 Questionnaires/ (429592)
63 Focus Groups/ (27030)
64 Health Surveys/ (59635)
65 Health Care Surveys/ (30884)
66 Interviews as Topic/ (57899)
67 (questionnaire$ or survey$).tw. (956462)
68 (focus adj3 group$).tw. (42736)
69 exp Reminder Systems/ (3237)
70 remind$.tw. (18000)
71 Telephone/ (11288)
72 telephone.tw. (54222)
73 phone call.tw. (1179)
74 Health Behavior/ (47125)
75 Behavior Therapy/ (26940)
76 (behavioral or behaviour$).tw. (521647)
77 (increas$ adj3 (attend$ or uptake)).tw. (33089)
78 (approachability or acceptability or availability or affordability or appropriateness).tw. (248806)
79 (ability adj2 (perceive or seek or reach or pay or engage)).tw. (3494)
80 exp Vision Tests/ (99239)
81 Mass Screening/ (98002)
82 (vision adj3 (test$ or screen$ or assess$)).tw. (4649)
83 (eye$ adj3 (test$ or screen$ or assess$)).tw. (8507)
84 ((target$ or tailor$) adj3 intervention$).tw. (48273)
85 (cultural$ adj3 (sensitiv$ or appropriate)).tw. (9151)
86 or/48-85 (3037015)
87 18 and 47 and 86 (4539)
88 exp developing countries/ (72422)
89 87 not 88 (4463)
90 prevalence.ti. (123792)
91 89 not 90 (3984)
92 exp case report/ (2028133)
93 (case$ adj3 (report$ or series)).tw. (739059)
94 92 or 93 (2268229)
95 91 not 94 (3572)
96 chi.lg. (313127)
97 95 not 96 (3550)
98 limit 97 to yr="1990 -Current" (3275)

Table S1.2. *Grey Literature Search Strategy and Selection*

All searches took place on 22/07/2021, and all resources were screened, selected and data was extracted between 22/07/2021 and 30/07/2021.

	SEARCH SOURCE AND STRING	NO. OF HITS	NO. IDENTIFIED FOR SCREENING	NO. IDENTIFIED FOR FULL TEXT REVIEW	NO. INCLUDED FOR EXTRACTION
	**Google Advanced Searches**				
1	“ethnic minority” AROUND(3) eyecare OR eye OR "visual impairment" OR cataract OR "refractive error" OR "diabetic retinopathy" OR glaucoma	39	5 (checked all)	4	4
2	“ethnicity” AROUND(3) eyecare OR eye OR "visual impairment" OR cataract OR "refractive error" OR "diabetic retinopathy" OR glaucoma	77	1 (checked all)	1	0
3	“non-dominant” AROUND(3) eyecare OR eye OR "visual impairment" OR cataract OR "refractive error" OR "diabetic retinopathy" OR glaucoma	>100	0 (checked 1st 20)	0	0
4	“Black” AROUND(3) eyecare OR eye OR "visual impairment" OR cataract OR "refractive error" OR "diabetic retinopathy" OR glaucoma	>100	0 (checked 1st 20)	0	0
5	“African American” AROUND(3) eyecare OR eye OR "visual impairment" OR cataract OR "refractive error" OR "diabetic retinopathy" OR glaucoma	>100	6 (checked 1st 50)	6	0
6	“Latinx” AROUND(3) eyecare OR eye OR "visual impairment" OR cataract OR "refractive error" OR "diabetic retinopathy" OR glaucoma	59	3 (checked all)	3	1
7	“Latino” AROUND(3) eyecare OR eye OR "visual impairment" OR cataract OR "refractive error" OR "diabetic retinopathy" OR glaucoma	>100	13 (checked 1st 50)	6	2
8	“Hispanic” AROUND(3) eyecare OR eye OR "visual impairment" OR cataract OR "refractive error" OR "diabetic retinopathy" OR glaucoma	>100	6 (checked 1st 50)	6	3
9	“Pacific” AROUND(3) eyecare OR eye OR "visual impairment" OR cataract OR "refractive error" OR "diabetic retinopathy" OR glaucoma	72	0 (checked 1st 20)	0	0
10	“Asian” AROUND(3) eyecare OR eye OR "visual impairment" OR cataract OR "refractive error" OR "diabetic retinopathy" OR glaucoma	>100	0 (checked 1st 20)	0	0
11	“Indian” AROUND(3) eyecare OR eye OR "visual impairment" OR cataract OR "refractive error" OR "diabetic retinopathy" OR glaucoma	>100	0 (checked 1st 20)	0	0
12	“Improving access” AROUND(3) eyecare OR eye OR "visual impairment" OR cataract OR "refractive error" OR "diabetic retinopathy" OR glaucoma	>100	8 (checked 1st 50)	6	2
13	“Improve access” AROUND(3) eyecare OR eye OR "visual impairment" OR cataract OR "refractive error" OR "diabetic retinopathy" OR glaucoma	>100	6 (checked 1st 50)	2	0
14	“Equity” AROUND(3) eyecare OR eye OR "visual impairment" OR cataract OR "refractive error" OR "diabetic retinopathy" OR glaucoma	>100	2 (checked 1st 50)	1	0
15	“Intervention improve access” AROUND(3) eyecare OR eye OR "visual impairment" OR cataract OR "refractive error" OR "diabetic retinopathy" OR	0	(adds only)	0	0

	glaucoma				
	**Grey Literature databases**				
	**Grey Lit**				
16	“ethnicity” AND (eyecare OR eye OR "visual impairment" OR cataract OR "refractive error" OR "diabetic retinopathy" OR glaucoma)	0	0	0	0
17	eyecare	10	0	0	0
	**Guidelines International Network ‘GIN’**				
18	“ethnicity” AROUND(3) eyecare OR eye OR "visual impairment" OR cataract OR "refractive error" OR "diabetic retinopathy" OR glaucoma	0	0	0	0
19	“ethnicity” AND (eyecare OR eye OR "visual impairment" OR cataract OR "refractive error" OR "diabetic retinopathy" OR glaucoma)	0	0	0	0
20	eyecare	0	0	0	0
21	"eye care”	2	0	0	0
	**Open Grey**				
22	“ethnicity” AROUND(3) (eyecare OR eye OR "visual impairment" OR cataract OR "refractive error" OR "diabetic retinopathy" OR glaucoma)	0	0	0	0
23	“ethnicity” AND (eyecare OR eye OR "visual impairment" OR cataract OR "refractive error" OR "diabetic retinopathy" OR glaucoma)	3	0	0	0
24	access to eyecare ethnic minorities	0	0	0	0
25	eyecare	0	0	0	0
26	"eye care"	4	1	0	0
	**Trip Database**				
27	“ethnicity” AROUND(3) (eyecare OR eye OR "visual impairment" OR cataract OR "refractive error" OR "diabetic retinopathy" OR glaucoma)	0	0	0	0
28	“ethnicity” AND (eyecare OR eye OR "visual impairment" OR cataract OR "refractive error" OR "diabetic retinopathy" OR glaucoma)	0	0	0	0
29	access to eyecare ethnic minorities	0	0	0	0
30	eyecare AND ethnicity	4	0	0	0
	**Total Extraction**				12 *(all from google searches)*

***Table S1.3**. Websites used for data extraction after grey lit search*

Resources	Initiative(s)	Year *Target population* Target condition *Country*	Approach
http://news.bbc.co.uk/2/hi/uk_news/scotland/glasgow_and_west/7544704.stm<br>https://www.opticianonline.net/news/minorities-targeted	Glasgow (equity project)	2008 *Mixed ethnicities* General eyecare *UK*	Few details
https://sightcymru.org.uk/launch-of-a-campaign-drive-to-engage-black-and-ethnic-minority-communities-with-eye-care-at-the-pierhead-cardiff-30th-november-2015/<br>https://business.senedd.wales/mgCalendarEvent.aspx?Id=1537&RPID=0	Cardiff (equity project)	2015 *Mixed ethnicities* General eyecare *UK*	Education and screening programme
https://ec.europa.eu/eip/ageing/news/promoting-well-being-and-improving-access-eye-care_en.html	"P5SE" by Tays Eye Centre	2016 *Mixed ethnicities* General eyecare *Finland*	'P5SE' Prioritise 'worst outcome' eye diseases, Stratify, based on outcomes, Standardise care pathways, Streamline decision processes, Shared care using multidisciplinary teams, ensure services are Sustainable, and embed continuous Evaluation
https://www.nei.nih.gov/learn-about-eye-health/outreach-campaigns-and-resources/eye-health-among-hispanicslatinos	National Eye Institute (NEI), part of National Institute of Health (NIH)	2019 *Latinx* General eyecare *USA*	Targeted awareness and educational materials
https://cao.memberclicks.net/assets/docs/prioritizing_eye_health_in_hispanic_communities.pdf	Transitions Cultural Connections	NA *Latinx* General	In depth recommendations for

	, National Association of Hispanic Nurses	eyecare *USA*	patients and providers, covering many aspects of Levesque access framework
http://bdweb8960p.bluedomino.com/pdf/April2018.pdf	Health Plan San Joaquin eyecare initiatives	2018 *Latinx* General eyecare *USA*	Advertising free eyecare services in local, Spanish newspaper
https://mdnewsline.com/latinx-patients-more-affected-by-diabetic-macular-edema/	Affordable Care Act Ventanillas de Salud (VDS) Juntos por la Salus (JPLS)	2020 *Latinx* General eyecare *USA*	Variety of resources for diabetes
https://www.glaucoma.org/gleams/hispanic-americans-at-increased-risk-for-glaucoma.php	National Eye institute (NEI) EyeCare America (*American Academy of Ophthalmology*) Glaucoma Research Foundation	2017 *Mixed ethnicities* General *USA*	Variety of resources for glaucoma
https://www.healio.com/news/ophthalmology/20120331/high-glaucoma-prevalence-in-blacks-leaves-questions-about-treatment	Travatan Project Focus (*Alcon*) Partnership for Sight Initiative *(Allergan)* Glaucoma Eyecare Program (*American Academy of Ophthalmology*)	2003 *Black* Glaucoma *USA*	Variety of resources for glaucoma. Most including educational and screening components
https://www.iapb.org/blog/improving-access-to-glaucoma-care-a-population-health-priority/	Screening and Intervention for Glaucoma and Eye Health through Telemedicin	2021 *Mixed ethnicities* Glaucoma *USA*	Tele-screening, and follow-up support

	e (SIGHT) Program		
