# Supplementary material for: Interventions to promote access to eyecare for non-dominant ethnic groups in high-income countries: a scoping review
Source: BMJ Glob Health. 2021 Sep 7;6(9):e006188. doi: 10.1136/bmjgh-2021-006188 (PMC8424858; doi:10.1136/bmjgh-2021-006188)
Supplement: Supplementary data [file bmjgh-2021-006188supp002.pdf]

Supplementary File 2

***Table S2.1**. Studies which were not evaluated*

First Author	Target Condition	Target Population	Colab Design	Intervention description	Levesque side prioritised	Results
D'Lugoff, 2005 USA	Diabetic Retinopathy	Black (adults)	No	Community screening and education (n=93)	Provider	93 screened, 31 (33%) referred
Owsley, 2015 USA	Diabetic Retinopathy	Black (adults)	No	Telemedicine screening (n=1894)	Provider	22% referred for diabetic retinopathy, 31% cataract, 10% glaucoma
Winters, J, 2008 USA	General	Mixed ethnicities (adults)	No	Integrated services to help coordinate referral to treatment (n=2471)	Both	1753 attended appointments, of these: 86% needed and received refraction, 9% had cataracts, 2% glaucoma, 10% glaucoma (or ocular hypertension) suspect 8% diabetic retinopathy
Shahid, 2012 USA	General	Black (all ages)	No	Telemedicine screening and some treatment at soup kitchens (n=341)	Provider	6% referred for significant cataract, 10% for glaucoma, 2% diabetic retinopathy, 13% other vision threatening disease
Feldman, 1977 Canada	General	Asian (all ages)	No	Community assessments (n=235)	Both	235 screened, 94 ocular pathology (including refractive error: 45, Cataract: 25)
Sanspree, 2008 USA	General	Black (all ages)	Yes	Community screening treatment programme (n=2699)	Both	39% referred for refraction (56% spectacles), 8% for cataract, 19% for glaucoma, 2.7% for diabetic retinopathy
Winters, J, 2008 USA	General	Black (adults)	No	Comprehensive screening and follow-up program (n=89)	Both	All needed and received glasses, 39% referred for cataract, 23% for glaucoma, 15% diabetic retinopathy
MacLean, 2014 USA	General	Mixed ethnicities (adults)	No	Community eye exam program connected to training students (n=145)	Provider	26% refractive error 20% cataract, 10% glaucoma suspect, 8% diabetic retinopathy (+ many other specific diagnoses)
Preslan, 1996 USA	General (children)	Black (children)	No	Screening and treatment programme (n=680)	Both	11% failed, 8% had refractive errors
Kemper, 2004 USA	General (children)	Latinx (children)	No	Medicaid (n=441584)	Provider	9% accessed eyecare, 5% refractive services
Mehravaran, 2016 USA	General (children)	Latinx (children)	No	Screening and treatment programme (n=11260)	Provider	8% received refractive correction
Miller, 1976 USA	General (children)	Latinx (children)	No	Screening and treatment program (n=8900)	Provider	Referred: 11% (preschool), 20% school-aged
Kattouf, 2009 USA	General (children)	Mixed ethnicities (children)	No	Screening, treatment, and education (n=4298)	Both	17% received spectacles
Hark, 2018 USA	Glaucoma	Black (adults)	Yes	Comprehensive screening and follow-up program (n=182)	Both	182 screened, 108 (59%) referred, 89 (82%) attended follow-up
Kikut, 2020 USA	Glaucoma	Black (adults)	No	Various public health marketing campaigns (radio, TV, postcards, etc) *grouped data*	Patient	Commercials best, but most expensive, generally spend $400-$800 for each participant recruited
Al-Aswad, 2017 USA	Glaucoma	Latinx (adults)	No	Community screening (n=8547)	Provider	8547 screened, 2118 referred for Glaucoma, 1243 referred for ophthalmic exam
Vistamehr, 2006 USA	Glaucoma	Black (adults)	No	Screening and treatment programme (n=184)	Provider	Interested in tools for screening, found 81.7% sensitivity and 55% specificity for protocol
Dreer, 2016 USA	Glaucoma	Black (adults)	Yes	Education, motivational interviews, and problem-solving training, carried out by psychologist (n=11)	Patient	Pilot results look promising for further research
Monaghan, 2011 USA	Injury prevention	Latinx (adults)	Yes	Behavioural activation with CHWs to promote understanding and use of PPE	Patient	Interview and focus groups showed promising results

	(n=74 interviews)

***Table S2.2**. Studies with concurrent comparators*

First Author	Target Condition	Target Population	Colab Design	Levesque side prioritised	Intervention 1 description	Intervention 2 description	Intervention 3 description	Intervention 4 (if usual care)	Outcome	Results	Effective?
Tjiam, 2013 The Netherlands	General (children)	Mixed ethnicities (children)	No	Patient	Cartoon information (n=25)	Parent leaflet information (n=21)	Rewards and calendar (n=24)	Usual care (n=18)	Adherence	Intervention 1:89%, Intervention 2: 73%, Intervention 3: 67%, Usual care: 55%	Effective
Forst, 2004 USA	Injury prevention	Latinx (adults)	Yes	Patient	Provide free protective eyewear from CHW + CWH training (n=256)	Provide free protective eyewear + CHW training (n=298)	Free protective eyewear (n=149)	NA	Adherence	Intervention 1: best uptake of protective eyewear	Effective
Nesher, 2001 Israel	Glaucoma	Mixed ethnicities (adults)	No	Provider	Pre-recorded instructions (n=30)	Interpreter-assisted instructions (n=30)	NA	NA	Adherence	No difference between groups (authors conclude either is an effective option)	Inconclusive
Monaghan, 2012 USA	Injury prevention	Latinx (adults)	Yes	Patient	Free protective eyewear from CHW + CWH peer modelling/training *(grouped data)*	Free protective eyewear *(grouped data)*	NA	NA	Adherence	CHW improved use of protective eyewear (note measured by 'crews' not individuals)	Inconclusive
Tovar-Aguilar, 2014 USA	Injury prevention	Latinx (adults)	Yes	Both	Free protective eyewear, eye drops + training *(grouped data)*	NA	NA	Usual practice on farm *(grouped data)*	Adherence	Some promising results (particularly qualitative and pre/post)	Inconclusive
Prezio, 2014, USA	Diabetic Retinopathy	Latinx (adults)	No	Patient	CHW support (7 hours) (n=90)	NA	NA	Usual care (n=90)	Eye exam	Intervention: 47%, Usual care: 33%	Effective
Davis, 2003 USA	Diabetic Retinopathy	Black (adults)	No	Provider	Telemedicine screening (n=30)	NA	NA	Usual care (n=29)	Eye exam	Intervention: 77%, Usual care: 14%	Effective
Bush, 2014 USA	Diabetic Retinopathy	Asian (adults)	No	Patient	Link Worker–implemented telephone call (n=988)	NA	NA	Usual care (n=1692)	Eye exam	Intervention: 89%, Usual care: 74%	Effective
Anderson, 2003 USA	Diabetic Retinopathy	Black (adults)	Yes	Patient	Personalised supportive reminder calls (n=67)	NA	NA	Usual care (n=65)	Eye exam	Intervention: 66%, Usual care: 35%	Effective
Basch, 1999 USA	Diabetic Retinopathy	Black (adults)	No	Patient	Multicomponent supportive educational intervention (booklet, video, semi structured telephone education and counselling) (n=137)	NA	NA	Usual care (n=143)	Eye exam	Intervention: 55%, Usual care: 27%	Effective
Pizzi, 2015 USA	Diabetic Retinopathy	Black (adults)	No	Patient	Reminder + telephone information (n=120)	Reminder + mailed information (n=117)	NA	Usual care (n=119)	Eye exam	Intervention 1: 51%, Intervention 2: 32%, Usual care: 36%	Effective

Weiss, 2015 USA	Diabetic Retinopathy	Black (adults)	No	Patient	Behavioural activation (n=91)	Supportive therapy	NA	NA	Eye exam	Intervention 1: 88%, Intervention 2: 34%	Effective
Walker, 2008 USA	Diabetic Retinopathy	Mixed ethnicities (adults)	No	Patient	Personalised supportive reminder calls (n=326)	Information packages	NA	NA	Eye exam	Intervention 1: 34%, Intervention 2: 20%	Effective
Rodriguez, E, 2018 USA	General (children)	Latinx (children)	No	Both	School vision screening program + full time school nurse (n=2800)	School vision screening program (n=3445)	NA	NA	Eye exam	Intervention 1: 97%, Intervention 2: 67%	Effective
van Zyl	Glaucoma	Mixed ethnicities (adults)	No	Provider	Counselling + recommended follow up pre-scheduled (n=22)	Counselling + recommended follow up without pre-scheduling (n=41)	NA	NA	Eye exam	Intervention 1: 41%, Intervention 2: 24%	Effective
Zhang, 2009 USA	Diabetic Retinopathy	Black (adults)	Yes	Patient	DIRECT program + eyecare education *(grouped data)*	DIRECT program *(grouped data)*	NA	NA	Eye exam	Intervention 1: 73%, Intervention 2: 66% *(difference not significant)*	Inconclusive
Ellish, 2011 USA	General	Black (adults)	No	Patient	Tailored (individualized) print information (n=137)	targeted (designed for a subgroup) print intervention (n=142)	NA	NA	Eye exam	Intervention 1: 40%, Intervention 2: 38% *(those who read either were 1.8 times more likely to attend)*	Inconclusive
Hark, 2016 USA	Glaucoma	Black (adults)	Yes	Both	Follow-up eye care in a community-based setting with assistance from a patient navigator (n=53)	Follow-up eye care in an office-based setting with assistance from a patient navigator (n=57)	Follow-up eye care in an office-based setting without a patient navigator (usual care) (n=45)	NA	Eye exam	Intervention 1: 70%, Intervention 2: 83%, Intervention 3: 73% *(not significant; all follow-up attendance very good, and other metrics showed intervention 1 to be most effective)*	Inconclusive
Aleo, 2015 USA	Diabetic Retinopathy	Black (adults)	No	Patient	Sign contracts (n=42)	NA	NA	Usual care (n=41)	Eye exam	Intervention: 38%, Usual care: 44%	Ineffective
Meng, 2016 USA	Diabetic Retinopathy	Mixed ethnicities (adults)	No	Patient	Medicaid + disease management programme (n=2933)	NA	NA	Medicaid (n=2988)	Eye exam	Disparity in follow-up eye exams remained despite intervention (although it helped some non-dominant groups more than others)	Ineffective
Owsley, 2013 USA	General	Black (adults)	Yes	Both	InCharge group education (n=54)	Information on physical activity (n=63)	NA		Eye exam	Eye-specific training did not improve attendance *(No pre to post improvement on survey results either)*	Ineffective
Welch, 2011 USA	Diabetic Retinopathy	Latinx (adults)	Yes	Both	Interactive web-based diabetes management tool (n=21)	Attention control (n=18)	NA	NA	Health	More improvement in A1c in Intervention 1	Effective

Vaughan, 2017 USA	Diabetic Retinopathy	Latinx (adults)	No	Both	CHW lead group education/support/behavioural activation (n=25)	NA	NA	Usual care (n=25)	Health	Group sessions improved target A1c levels, and screening rate (Intervention: 57%, Usual care: 25% achieved target A1c levels)	Effective
Rovner, 2019 USA	Diabetic Retinopathy	Black (adults)	No	Provider	Culturally tailored behavioural health/ophthalmologic intervention called Collaborative Care for Depression and Diabetic Retinopathy (CC-DDR) (n=16)	NA	NA	Enhanced usual care (n=17)	Health	Difference was not significant between interventions, but whether patients trusted the ophthalmologist was an important factor	Inconclusive
Earle-Richardson, 2014 USA	Injury prevention	Latinx (adults)	Yes	Patient	Eye drops, protective eyewear, and an in-person presentation and pocket card on eye health (including how to use the eye drops and eyewear) (n=59)	NA	NA	Usual practice on farm (n=61)	Health	Eye pain decreased significantly, but redness, blurred vision, itch, and dry feeling did not	Inconclusive
Mehranbod, 2020 USA	Diabetic Retinopathy	Latinx (adults)	No	Patient	Automated reminder call (n=184)	NA	NA	Usual care (n=117)	Screened	intervention: 60%, control: 46%	Effective
Moussa, 2013 USA	Diabetic Retinopathy	Black (adults)	No	Patient	E-health interactive tutorials (n=23)	Print information (n=23)	NA	NA	Survey	Difference in knowledge not significant between interventions (but pre-post improved with both interventions)	Effective
Wagner, 2008 USA	Diabetic Retinopathy	Mixed ethnicities (adults)	No	Patient	Patient education (n=45)	NA	NA	Usual care (n=45)	Survey	Knowledge improved	Effective

***Table S2.3**. Studies with sequential comparators*

First Author	Target Condition	Target Population	Colab Design	Levesque side prioritised	Intervention description	Outcome	Results	Effective?
Tjiam, 2012 The Netherlands	General (children)	Mixed ethnicities (children)	No	Patient	Cartoon information (n=65)	Adherence	Several adherence-related metrics improved	Effective
Snipes, 2015 USA	Injury prevention	Latinx (adults)	No	Patient	Free protective eyewear + tailored reminders (n=41)	Adherence	Use improved	Effective
Luque, 2007 USA	Injury prevention	Latinx (adults)	Yes	Patient	Free protective eyewear + peer education + peer modelling of wear (n=427)	Adherence	Use and perceptions improved	Effective
Jenkins, C, 2004 USA	Diabetic Retinopathy	Black (adults)	Yes	Both	Integrated screening, treatment, empowerment, re-imbursement, and education program (n=270)	Eye exam	Pre: 21%<br>Post: 43%	Effective
Hong, 2019 USA	General (children)	Black (children)	No	Provider	Universal pre-kindergarten program (53,000 enrolled)	Eye exam	Diagnosis: improved by 55%<br>Treatment: improved by 45%<br>*(both from baseline)*	Effective
Zhao, 2018 USA	Glaucoma	Black (adults)	No	Both	STOP + Financial incentives, educational videos, race concordance with screeners, and making follow up appointments close to the time of screening	Eye exam	Pre: 47% (STOP only)<br>Post: 64% (STOP +)	Effective
Chedid, 2013 USA	Diabetic Retinopathy	Black (adults)	No	Provider	program + improved staff training, referral processes and patient reminder calls *(grouped data, compared to initial program)*	Eye exam	Pre: 20%<br>Post: 71%	Inconclusive
Draper, 2016 USA	General	Black (adults)	No	Provider	Home-based low vision services (n=24) and clinic-based services (n=24)	Health *(Self-report functional vision)*	Pre/post improvement *(no difference between home and clinic based)*	Effective
Baker, S, 1993 USA	Diabetic Retinopathy	Black (adults)	Yes	Both	Comprehensive program with patient management system (n=1164, 744 at 2nd time point)	Health *(Incidence of blindness)*	Pre: 9.5/1,000<br>Post: 2.7/1,000 *(across 4-years)*	Effective
Jani, 2017 USA	Diabetic Retinopathy	Black (adults)	No	Provider	Telemedicine screening (n=1661)	Screened	Pre: 25%<br>Post: 40%,	Effective
Lee, S, 2000 Australia	Diabetic Retinopathy	Mixed ethnicities (adults)	No	Both	Multi-faceted recruitment (print, radio, newsletter, etc) (n=1197)	Screened	Pre: 55%<br>Post: 70%	Effective
Olayiwola, 2011 USA	Diabetic Retinopathy	Mixed ethnicities (adults)	Yes	Provider	Telemedicine screening *(grouped data)*	Screened	Pre: 10-12%<br>Post: 20%,	Effective
Taylor, C, 2007 USA	Diabetic Retinopathy	Mixed ethnicities (adults)	No	Provider	Telemedicine screening and coordination of services (n=495)	Screened	Pre: 23%<br>Post: 59%	Effective
Navuluri, 2000 USA	Diabetic Retinopathy	Latinx (adults)	No	Patient	Community screening and education (n=19)	Survey	Knowledge on survey increased	Effective
DeNomie, 2019 USA	Diabetic Retinopathy	Latinx (adults)	Yes	Provider	Telemedicine screening (n=17)	Survey	Satisfaction high within focus groups	Effective
Frazier, 2012 USA	General (children)	Latinx (children)	Yes	Patient	Print information (n=42)	Survey	Knowledge improved	Effective
Kharod, 2006 USA	Glaucoma	Black (adults)	No	Provider	Translated, written instructions for glaucoma medication (n=164)	Survey	Knowledge of medication improved	Effective
Baker, H, 2008 USA	Glaucoma	Asian (adults)	No	Patient	Various public health education campaigns (television, local radio, local press, places of worship) (n=306)	Survey	Knowledge of glaucoma improved (radio most effective)	Effective
Rhodes, 2016 USA	Glaucoma	Black (adults)	No	Patient	Education program (EQUALITY) (n=651)	Survey	Knowledge and perceptions improved	Effective
Owsley, 2008 USA	General	Black (adults)	Yes	Both	InCharge group education (n=85)	Survey	Improvement not statically significant	Inconclusive

Table S2.4. *Papers associated with other studies*

First Author	Key or final paper in the study	Target Condition	Target Population	Levesque side prioritised	Intervention description	Results
Anderson, 2002 USA	Anderson, 2003	Diabetic Retinopathy	Black (adults)	Both	Host of culturally relevant interventions aimed at patient side of access (including behavioural activation)	Many barriers exist at a wider-systems and provider level, which are difficult to overcome
Jones, H, 2010 USA	Walker, 2008	Diabetic Retinopathy	Mixed ethnicities	Patient	Personalised reminder calls (n=305) further explored	Rapport is perhaps more important than race-concordance
Casten, 2011 USA	Weiss, 2015	Diabetic Retinopathy	Black (adults)	Patient	Early results from behavioural activation vs supportive therapy	Knowledge improved after intervention
Winters, D, 2017 USA		Diabetic Retinopathy	Black (adults)	Patient	Cost effectiveness comparison of behavioural activation vs supportive therapy	Behaviour activation costs more than supportive therapy, but behavioural activation is more effective, so better cost by outcome
Murchison, 2016 USA	Owsley, 2015	Diabetic Retinopathy	Mixed ethnicities (adults)	Provider	Community telemedicine screening (n=1914 across 4 sites)	25% - some level of diabetic retinopathy
Jani, 2017 USA	Jani, 2017	Diabetic Retinopathy	Black (adults)	Provider	Geographic information systems to link need with allocation of telemedicine screening services	Geographic information systems mapping can highlight areas of higher access needs
Zhao, 2017 USA	Zhao, 2018	Glaucoma	Black (adults)	Both	Comprehensive screening and follow-up program (n=901)	Ocular outcomes: 15% significant eye disease (including 8.7% glaucoma, 6.8% cataract), and 12% refractive error
Sapru, 2017 USA	Hark, 2016 and Rhodes, 2016	Glaucoma	Black (adults)	Both	Comprehensive screening and follow-up program	Detection and treatment rates: 38% glaucoma related diagnosis, 12% treated, 99% satisfaction
Hark, 2016 USA (PGDTP)	Hark, 2016	Glaucoma	Black (adults)	Both	Comprehensive screening and follow-up program (n=1649)	Education: Knowledge improved on survey (pre/post)
Waisbourd, 2016 USA (PGDTP)		Glaucoma	Black (adults)	Both	Comprehensive screening and follow-up program (n=1649)	Detection and treatment rates: 38% glaucoma related diagnosis, 12% treated, 99% satisfaction
Zheng, C, 2016 USA (PGDTP)		Glaucoma	Black (adults)	Both	Comprehensive screening and follow-up program (n=1649)	Barriers to access: lower than hoped screening numbers, and only 25% follow up.
Pizzi, 2018 USA (PGDTP)		Glaucoma	Black (adults)	Both	Comprehensive screening and follow-up program (n=1649)	Cost analysis: relatively small cost for number of issues identified
Adeghate, 2019 USA (PGDTP)		Glaucoma	Black (adults)	Both	Comprehensive screening and follow-up program (n=249)	Glaucoma detection and treatment: 249 completed eye exam, 90 attended follow up, 47 glaucoma related diagnosis (20 treated with drops, 26 treated with laser therapy)
Hark, 2019 USA (PTGDF)	Key results not out at time of writing *(*Hark, 2018 reported as 'study')*	Glaucoma	Black (adults)	Both	Comprehensive screening and follow-up program (n=906)	Diagnostic confirmation: 906 screened, 536 invited to and 347 attended second visit (same location).
Hark, 2020 USA (PTGDF)		Glaucoma	Black (adults)	Both	Comprehensive screening and follow-up program (n=347)	Cataracts: 906 screened, 347 completed follow up, 267 had cataracts, 38 visually significant
